# ICT penetration and life expectancy in emerging market economies: panel evidence from asymmetric causality analysis

**DOI:** 10.1186/s12889-024-18323-1

**Published:** 2024-03-26

**Authors:** Yilmaz Bayar, Ahmet Ozen, Mahmut Unsal Sasmaz, Marina Danilina

**Affiliations:** 1 Bandirma Onyedi Eylul University, Balikesir, Türkiye; 2 Dokuz Eylul University, Izmir, Türkiye; 3 Usak University, Usak, Türkiye; 4 Plekhanov Russian University of Economics, Moscow, Russia; 5 Financial University under the Government of the Russian Federation, Moscow, Russia

**Keywords:** Mobile subscription, Internet usage, ICT penetration, Life expectancy, Public health, Sustainable development, Emerging market economies, Asymmetric causality analysis

## Abstract

**Background:**

Life expectancy is a significant result indicator of public health and sustainable development. Therefore, one of the final objectives of all economic and social policies is to increase the life expectancy. In this context, a limited number of researchers have investigated the relationship between ICT penetration and life expectancy. However, multiple interaction channels exist between ICT penetration and life expectancy. Furthermore, the studies have usually focused on the effect of ICT penetration on life expectancy through regression and ignored the effect of life expectancy on ICT penetration to a large extent. Therefore, this study aims to contribute to the empirical literature by investigating the causal relationship between ICT indicators and life expectancy.

**Methods:**

This study uses symmetric and asymmetric causality approaches to investigate the two-way interaction between ICT indicators and life expectancy in emerging market economies over the 1997–2020 period. Employment of the asymmetric causality test enables us to analyze the hidden relationships between ICT indicators and life expectancy, unlike the traditional causality test.

**Results:**

The results of the symmetric causality test uncover a bidirectional causal interaction between mobile subscriptions and life expectancy but a one-way causal relationship from life expectancy to internet usage. However, the asymmetric causality test results uncover a unidirectional causal relationship between mobile subscriptions and life expectancy in China, Colombia, Czechia, Egypt, Greece, India, Kuwait and Turkiye due to positive shocks from mobile subscriptions. On the other hand, a bidirectional causal interaction exists between internet usage and life expectancy in all countries due to negative shocks from internet usage and life expectancy. Last, a unidirectional causal relationship exists between internet usage and life expectancy in all countries due to positive shocks from internet usage.

**Conclusion:**

ICT indicators significantly influence life expectancy health in a sample of emerging market economies. Therefore, internet usage and mobile devices are significant tools to improve life expectancy.

## Introduction

Life expectancy is a crucial result indicator of public health, well-being, and economic policies [1] and also one of the 17 Sustainable Development Goals (S.D.G.s) entitled “Ensure healthy lives and promote well-being for all at all ages” (SDG-3) [2]. In this regard, life expectancy is a significant indicator of human development and population health [3, 4]. Furthermore, the achievement of the other S.D.G.s is closely related to public health because human capital can contribute more to the economy through innovation, technological progress, and production as life expectancy increases [5]. The leading countries in terms of human development, such as Japan, the United States of America, Singapore, South Korea and Germany, have a high capacity to produce new technologies, and these countries are the pioneers of the digital world [6, 7]. Therefore, every country tries to experience improvements in public health through economic growth and development, education, and health policies.

The average life expectancy in the world was 30 years before industrialization but has reached 70 years in parallel with the developments in the health sector and technologies [8–10]. Improvements in medical and production technologies can contribute to life expectancy by diagnosing and treating diseases, sustainable economic growth, environmental sustainability, and green energy [11–14]. However, there has been remarkable heterogeneity in life expectancy among countries. For example, life expectancy at birth in Chad, Nigeria, and Lesotho were respectively 52.53, 52.68, and 53.06 in 2021, but life expectancy in Japan, Australia, and Switzerland were respectively 84.78, 84.53, and 83.99 in 2021 [15].

Therefore, identifying factors underlying heterogeneity in life expectancy is vital for optimal policy-making. In this context, the researchers have suggested improvements in the healthcare field and various socioeconomic factors such as income level, economic stability, unemployment, education, technological development, urbanization, forestry, and demographic factors [16–28]. Considering the limited empirical literature, this study investigates the interplay between information and communication technologies (ICT) and life expectancy.

In the literature, a limited number of research on the effect of ICT indicators, including Internet, mobile subscriptions, and fixed broadband subscriptions, on life expectancy in samples of different country groups, as seen in Table 1, and the studies have mainly discovered a positive effect of ICT indicators on life expectancy [29–39]. But Ilikkan Özgür et al. [40] uncovered a negative effect of mobile users and Internet subscribers on life expectancy in the short and long term in a sample of BRICS-T countries, Wang et al. [41] revealed a positive effect of mobile internet use and mobile cellular subscriptions on life expectancy and a negative effect of fixed telephone subscriptions on life expectancy in selected low-income states. Lastly, Vaidean and Achim [42] revealed an inverted U-shaped interaction between ICT indicators and life expectancy in a panel of 185 countries. In this context, the researchers have generally focused on the effect of ICT indicators on life expectancy through regression analysis and ignored the effect of life expectancy on ICT penetration. However, there can be feedback between ICT and life expectancy. Furthermore, the researchers have usually employed symmetric econometric methods to investigate the nexus between ICT and life expectancy.

In conclusion, this article aims to contribute to the empirical literature in three aspects. Unlike the related literature, the first empirical contribution of the study is to conduct a two-way analysis between ICT indicators and life expectancy. The second contribution of the study is to employ both asymmetric and traditional causality tests simultaneously to analyze the nexus between ICT and life expectancy. The third contribution of the paper is to analyze the nexus between ICT and life expectancy in the sample of emerging markets. Consequently, the findings of the study will be useful to arrange the policies toward improvements in public health.

The emerging markets are specified as the sample of the study because the emerging markets, including China, India, Indonesia, the Korean Republic, and Thailand, have been the drivers of global economic growth, global population, and ICT development and include the most innovative companies in the world [43]. All emerging economies except Mexico experienced varying degrees of improvements in life expectancy at birth. India, the Korean Republic, China, Thailand, and the United Arab Emirates obtained the most significant improvement in life expectancy at birth during the study period. But Kuwait, Greece, Indonesia, and the Philippines had the lowest improvement in life expectancy at birth [44]. However, all emerging countries experienced remarkable increases in internet usage and mobile cellular subscriptions during the study period [45, 46].

The next part of the article presents an extensive theoretical and empirical literature summary about the implications of ICT penetration; then, the dataset and econometric tests are described; econometric tests and discussion are introduced, and the article eventuates in the Conclusion.

## Theoretical background and empirical literature review

ICT can affect life expectancy through different direct and indirect aspects. In this context, ICT can foster life expectancy through increasing access to information and sharing about health, healthy nutrition, and epidemics [32, 47–49]. Online health information can also enhance individuals’ health-related knowledge, improve doctor-patient communication, and, in turn, increase the early detection and treatment of diseases and lead individuals to make informed decisions about life quality [50–52]. Furthermore, ICT increases clinic time’s effective and efficient use [53].

ICT penetration can also negatively affect life expectancy through obesity, heart disease and musculoskeletal system problems as a result of reducing the physical movements of the individuals [42, 54, 55]. ICT penetration may also negatively affect life expectancy through health problems such as severe obesity, back pain and neck pain, orthopaedic/joint muscles, eye problems, hearing problems and physical inactivity [56]. On the other hand, ICT can impact life expectancy through economic growth, financial development, unemployment, green energy development, energy use, electronic waste, innovation, entrepreneurship, and production [34, 57–74]. Therefore, a significant impact of ICT on life expectancy is expected a priori. However, improvements in life expectancy can also foster ICT because people have more time to use and develop the ICT. Therefore, a mutual or one-way interaction between ICT and life expectancy is possible in theoretical terms based on countries’ characteristics.

The nexus between ICT indicators and life expectancy has begun to be questioned mainly since 2019 even though multiple theoretical interaction channels exist between ICT indicators and life expectancy. Most of the empirical studies in Table 1 usually analyzed the effect of ICT indicators on life expectancy. They uncovered a positive effect of ICT indicators on life expectancy in countries with different development levels [29–39]. However, Wang et al. [41] revealed both positive and negative effects of ICT indicators on life expectancy in 28 low-income countries. Furthermore, Ilikkan Özgür et al. [40] disclosed a negative effect of mobile users and internet subscribers on life expectancy in short and long-term BRICS-T countries. Last, Vaidean and Achim [42] uncovered an inverted U-shaped interaction between ICT indicators and population health in a panel of 185 countries.

In the related literature, only Rahman and Alam [37] investigated the causality between ICT indicators and life expectancy and disclosed a unidirectional causal relationship between ICT and life expectancy. However, most empirical studies have disregarded the possible effect of life expectancy on ICT development to a great extent until now. The researchers have usually applied regression to analyze the nexus between ICT indicators and life expectancy, and in turn, country-level analysis has been ignored. Furthermore, the researchers have generally employed symmetric econometric approaches in the empirical analyses. Therefore, this study investigates the causal interplay between ICT indicators and life expectancy through symmetric and asymmetric causality tests at panel and country levels.

**Table 1:** Empirical literature summary on the nexus between ICT indicators and life expectancy

Study	Sample; study period	Method	Main findings
Mithas et al. [29]	61 countries; 2005	Regression	A positive effect of information technology investments on male and female life expectancy
Mimbi and Bankole [30]	27 African countries;	Data envelopment analysis, cluster analysis, and regression	A positive effect of ICT proxied by annual telecom investment, line capacity of exchanges, International internet bandwidth, and full-time telecoms staff on life expectancy
Lee and Kim [31]	16 Asian countries; 2009–2014	Regression	A positive effect of ICT indicators (broadband, mobile phone, and Internet security) on life expectancy
Majeed and Khan [32]	184 countries; 1990–2014	Regression	A positive effect of ICT indicators on life expectancy
Alzaid et al. [33]	156 countries; 1999, 2005, and 2010	Regression	A positive effect of the Internet on life expectancy
Shao et al. [34]	141 countries; 2012–2016	Regression	A positive effect of ICT indicators on public health
Ronaghi [35]	Middle Eastern countries; 2008–2018	Regression	A positive effect of ICT on life expectancy
Mlambo et al. [36]	SADC states: 2000–2018	Cointegration and regression analysis	A weak positive effect of mobile cellular telephone subscriptions on maternal health
Rahman and Alam [37]	Australia; 1990–2018	ARDL approach	A positive effect of ICT on life expectancy and unidirectional causal relationship from ICT to life expectancy
Zhang et al. [38]	12 Asian countries; 1991–2019	Dynamic ordinary least square and fully modified least squares	A positive effect of the Internet on life expectancy
Byaro et al. [39]	48 sub-Saharan Africa countries; 2000–2020	Quantile regression	A positive effect of Internet use health outcomes
Ilikkan Özgür et al. [40]	BRICS-T countries; 1990–2018	ARDL	A negative effect of mobile users and Internet subscribers on short- and long-term life expectancy.
Wang et al. [41]	28 countries, 2000–2017	Regression	A positive effect of mobile internet use and mobile cellular subscriptions on life expectancy and a negative effect of fixed telephone subscriptions on life expectancy
Vaidean and Achim [42]	185 countries; 2005–2018	Regression	An inverted U-shaped interaction between ICT indicators and population health

In the literature, the nexus between ICT and human development, which also consists of life expectancy, has been investigated by relatively more researchers, and these studies generally uncovered a positive relationship between ICT indicators and human development [75–81]. However, the developed countries reached a significant saturation due to their high technology and ICT investments. In contrast, ICT investments in other country groups caused significant improvements in education and health and, in turn, contributed more to human development [75].

The following two hypotheses will be tested in the research article depending on the related theoretical and empirical literature:

- **H1.** There is a significant association between internet usage and life expectancy.
- **H2.** There is a significant association between mobile cellular subscriptions and life expectancy.

## Methods

### Data

Through symmetric and asymmetric causality tests, this study investigates the two-way interaction between ICT indicators and life expectancy in 23 emerging market economies. The variables employed in the econometric analyses are displayed in Table 2. Life expectancy (LIFEXP) is represented by life expectancy at birth because nearly all studies in Table 1 represented life expectancy by life expectancy at birth, and data on life expectancy at birth was obtained from UNDP [44]. On the other hand, ICT is represented by two indicators (internet usage and mobile cellular subscriptions) considering Lee and Kim [31], Zhang et al. [38], Byaro et al. [39], Ilikkan Özgür et al. [40], Wang et al. [41]. Internet usage (INTERNET) is proxied by individuals using the Internet (% of the population). Mobile cellular subscriptions (MOBIL) are represented by mobile cellular subscriptions (per 100 people), and both ICT indicators are respectively obtained from World Bank [45 & 46].

**Table 2:** Data description

Variable abbreviation	Data definition	Data source
LIFEXP	Life expectancy at birth (years)	UNDP [44]
INTERNET	Individuals using the Internet (% of total population)	World Bank [45]
MOBIL	Mobile cellular subscriptions (per 100 people)	World Bank [46]

The study sample consists of 23 emerging markets (Brazil, Chile, China, Colombia, Czechia, Egypt, Greece, Hungary, India, Indonesia, Korea, Kuwait, Malaysia, Mexico, Peru, Philippines, Poland, Qatar, Saudi Arabia, South Africa, Thailand, Turkey, and the United Arab Emirates) and study term is 1997–2020 because sample size and period are optimized during this period considering the presence of ICT indicators. The Stata 17.0 and Gauss 12.0 are employed for econometric analyses.

The average life expectancy, internet usage as a per cent of the population, and mobile subscriptions per 100 people in emerging market economies are respectively 73.897 years, 37.361%, and 81.119. Still, both ICT penetration indicators show significant variation in the study sample as seen in Table 3.

**Table 3:** Main characteristics of the series

Variables	Obs	Mean	Std. Dev.	Min	Max
LIFEXP	552	73.897	4.966	53.9797	83.6557
INTERNET	552	37.361	29.543	0.0323	100
MOBIL	552	81.119	51.981	0.0879	221.3088

### Methodology

The causality between life expectancy and ICT indicators is respectively tested by Juodis-Karavias-Sarafidis (JKS) [82] causality test and Yılancı and Aydın [83] asymmetric causality test. The asymmetry refers to a variable with different responses to positive and negative shocks. Therefore, disregarding the asymmetric interaction between two variables can reduce the reliability of the empirical findings. In other words, the asymmetric causality test enables us to investigate the hidden relationship between two variables differently from the symmetric causality tests [83]. Consequently, employing the asymmetric causality test and the JKS (82) causality test would cause us to obtain more robust and reliable results.

JKS [82] causality test is developed for both homogenous and heterogeneous panels. Furthermore, the test employs the H.P.J. (Half-Panel Jacknife) technique by Dhaene and Jochmans [84] to decrease the pooled estimator’s Nickell bias. Last, the JKS [82] causality test generates relatively more reliable results in the case of T < N when compared with the Dumitrescu and Hurlin [85] causality test. The test is based on the following equation [82]:

1 $$y_{it}\;=\;\pi_{oi}+\sum\nolimits_{k=1}^k\delta_{pi}y_{i,t-k}+\sum\nolimits_{q=1}^Q\varphi_{qi}X_{i,t-k}+\varepsilon_{it}$$

for country i = 1,….N and years t = 1,…T.

In Eq. (1), ${X}_{i,t}$ is a scalar, ${\delta }_{p}$; I correspond to heterogeneous autoregressive effects and ${\phi }_{q,}$ I heterogeneous Granger causality effects. JKS [82] accepts that ${y}_{i,t-k}$ indicates an autoregressive distributed lag process under the null hypothesis, ${\phi }_{qi}=0$ for all I and q. This approach allows for a pooled estimator. To treat the bias problem of a pooled estimator, the test applies an H.P.J. estimator. When cross-sectional dependence occurs in panel data, the variance of the H.P.J. estimator can be obtained through bootstrapping. The obtained estimations are bias-corrected and give Wald statistics for the Granger non-causality test.

Yılancı and Aydın [83] improved the Kónya [86] bootstrap causality test regarding cross-sectional dependency and heterogeneity in a way that includes the asymmetric approach of Hatemi, J [87]. . Thus, Yılancı and Aydın [83] asymmetric causality test investigates how positive and negative shocks within the variables influence each other, unlike Kónya [86] bootstrap Granger symmetric causality test. As a result, Yılancı and Aydın [83] asymmetric causality test can uncover significant causal relationships that may be overlooked when a symmetric causality test is conducted. Therefore, this article performs an asymmetric causality test together with the JKS [82] symmetric causality test.

## Results

In the applied part of the article, pre-tests of cross-sectional dependence and heterogeneity are performed in the first step. In line with this objective, L.M., LM CD, and LM_adj_. Tests respectively by [87–90] are implemented, and the results of these tests are introduced in Table 4. The null hypothesis (H0 = cross-sectional independence) is declined at a 5% significance level, and cross-sectional dependency among the series is unveiled.

**Table 4:** Cross-sectional dependence tests’ results

Test	Test Statistic	Prob.
LM	1208	0.000
LM CD	17.22	0.0000
LM adj	104.8	0.0173

The homogeneity is investigated by Pesaran and Yamagata [91] in the second step, and the results of two homogeneity tests are introduced in Table 5. The null hypothesis in favour of homogeneity is declined at a 1% significance level, and heterogeneity is unveiled. In conclusion, unit root and causality tests that notice heterogeneity and cross-sectional dependence should be preferred for relatively more robust results.

**Table 5:** Homogeneity tests’ results

Test	Test Statistic	Prob.
$\tilde \Delta$	25.019	0.000
${\tilde \Delta _{{\text{adj}}{\text{.}}}}$	27.407	0.000

The stationarity analysis of three variables under consideration (LIFEXP, INTERNET, and MOBIL) is conducted by Pesaran [92] CIPS unit root test and test results are introduced in Table 6. LIFEXPT, INTERNET, and MOBIL are nonstationary for their level values but become stationary for their first-differenced values.

**Table 6:** CIPS panel unit root test results

Variables	Constant	Constant + Trend
LIFEXP	-0.878	1.074
d(LIFEXP)	-4.558^a^	-3.478^a^
INTERNET	1.912	0.874
d(INTERNET)	-2.606^a^	-5.785^a^
MOBIL	-0.201	0.592
d(MOBIL)	-6.382^a^	-5.076^a^

^a^it is significant at 1%

**Fig. 1:**
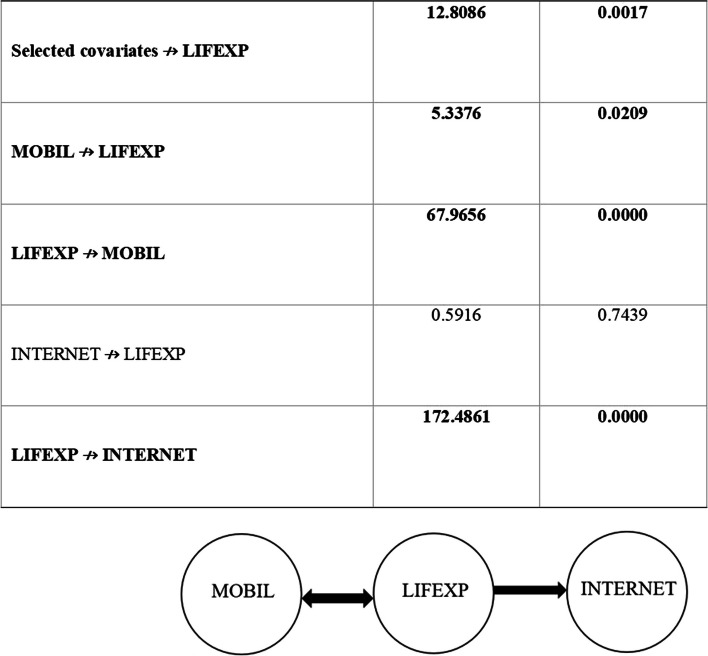
Results of JKS (2021) Granger non-causality test

The causal interaction between ICT indicators and life expectancy in 23 emerging market economies over the 1997–2020 duration is first investigated by the JKS [82] causality test. First, we test whether the pair of internet usage and mobile subscription Granger causes life expectancy and the results of the causality analysis are reported in Table 7. The null hypothesis that internet usage and mobile subscriptions do not Granger-cause life expectancy is rejected at the 5% significance level. Therefore, both indicators have a significant effect on life expectancy. Furthermore, univariate causality analyses uncover a bidirectional causality between mobile subscriptions and life expectancy and unidirectional causality from life expectancy to internet usage (Fig. 1).

**Table 7:** Results of JKS (2021) Granger non-causality test

**Null Hypothesis**	**H.P.J. Wald test**	***P* values**
**Selected covariates ↛ LIFEXP**	**12.8086**	**0.0017**
**MOBIL ↛ LIFEXP**	**5.3376**	**0.0209**
**LIFEXP** ↛ **MOBIL**	**67.9656**	**0.0000**
INTERNET ↛ LIFEXP	0.5916	0.7439
**LIFEXP** ↛ **INTERNET**	**172.4861**	**0.0000**

In the second stage, the causal interaction between ICT indicators and life expectancy is investigated through Yılancı and Aydın [83] asymmetric causality test and test results are introduced in Tables 8, 9, 10 and 11. First, the causality between MOBIL and LIFEXP with negative shocks is tested, and the results in indicate that there is not a significant causal interaction between two variables in case of negative shocks from both variables.

**Table 8:** Results of asymmetric bootstrap Granger causality test

Countries	MOBIL ↛ LIFEXP (-)				LIFEXP ↛ MOBIL (-)			
	Wald. Stat.	Bootstrap Critical Values			Wald. Stat.	Bootstrap Critical Values		
		1%	5%	10%		1%	5%	10%
Brazil	388.8971	2937.4151	787.4613	552.5777	563.5642	9486.2285	1660.7556	855.1336
Chile	388.9004	2655.0983	781.2009	550.2563	563.4352	10070.7565	1594.9264	862.3846
China	388.9049	2900.3285	778.3647	552.2556	563.5057	11986.3285	1682.6789	872.9949
Colombia	388.8796	2970.8222	789.5381	554.0158	563.5173	10252.4378	1690.2909	867.3679
Czechia	388.8792	2910.6685	786.4420	552.1317	563.5621	11851.9597	1727.7265	872.8187
Egypt	388.8878	2878.5091	793.1906	554.7188	563.5399	12777.6202	1660.0664	873.5820
Greece	388.8189	3102.6383	792.6803	553.9396	563.5668	11348.4541	1564.5671	873.3831
Hungary	388.8917	2761.5404	790.1609	554.0315	563.3726	12779.6594	1609.4294	836.0589
India	388.8619	3211.0305	787.8686	554.1767	563.5595	8907.3437	1675.7690	851.9747
Indonesia	388.7641	3066.6162	778.6839	554.4559	563.5556	9734.7872	1557.8606	861.5773
Korea	386.7365	2696.8575	781.9746	553.5933	563.4490	10862.2061	1612.1050	873.6177
Kuwait	388.6602	2890.2376	786.1060	552.7161	561.0216	10611.0512	1464.6705	858.3265
Malaysia	388.8791	2756.2314	784.7640	551.0671	529.2750	10405.8363	1682.4749	875.3577
Mexico	388.8436	2900.1807	778.6548	552.0594	563.5562	10297.8391	1657.6748	863.0830
Peru	388.9031	2808.1440	781.0338	552.7557	563.5679	12518.0953	1560.3053	854.9077
Philippines	388.9019	2653.7516	785.9607	555.7683	563.5556	12888.4247	1799.3115	891.4478
Poland	388.9033	2985.1986	795.1066	555.1616	562.8334	12598.8952	1754.3697	873.7802
Qatar	388.8933	3143.2487	790.6556	557.3084	563.5188	11893.8877	1690.6627	871.2358
Saudi Arabia	388.8954	2981.2147	786.8719	554.6219	563.5420	13081.0593	1660.7881	873.3847
South Africa	388.9012	3015.6914	797.9976	557.0983	563.5488	12551.0995	1684.1720	872.7918
Thailand	388.9033	2802.3853	783.2525	551.8219	563.5618	12576.2108	1515.0646	859.9611
Turkiye	388.7990	2886.9215	783.7597	554.7944	563.5677	11847.1994	1688.7809	863.1096
United Arab Emirates	388.7218	3161.2723	788.1452	555.5234	563.5679	7694.2533	1629.4062	859.9142

Bootstrap critical values are obtained from 10.000 iterations

**Fig. 2:**
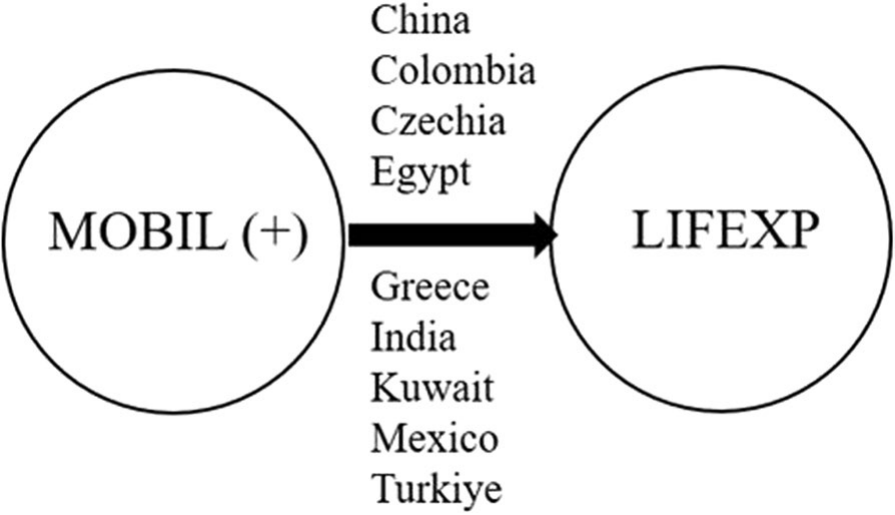
Results of asymmetric bootstrap Granger causality test between MOBIL and LIFEXP (+)

Secondly, the causality between MOBIL and LIFEXP with positive shocks is tested, and the results in Table 9 indicate a one-way significant causal relationship from MOBIL to LIFEXP in China, Colombia, Czechia, Egypt, Greece, India, Kuwait, and Turkiye in case positive shocks from MOBIL variable. In other words, a positive shock in MOBIL is a Granger cause of increases in LIFEXP (Fig. 2).

**Table 9:** Results of asymmetric bootstrap Granger causality test

Countries	MOBIL ↛ LIFEXP (+)				LIFEXP ↛ MOBIL (+)			
	Wald. Stat.	Bootstrap Critical Values			Wald. Stat.	Bootstrap Critical Values		
		1%	5%	10%		1%	5%	10%
Brazil	565.4367	3812.3196	943.1240	604.3860	355.6662	51619.1036	1406.9773	843.8296
Chile	566.0207	3642.9238	947.0138	601.5231	355.6657	4313.4299	1164.7782	763.1566
**China**	598.9844*	4841.8460	947.0978	595.3894	355.6657	4924.9710	1111.9786	806.4073
**Colombia**	565.9841*	3722.0261	976.0668	597.1208	355.6657	1785.5940	1078.0439	807.7678
**Czechia**	565.9843*	3492.5442	943.6050	579.6512	355.6656	14273.0168	1408.8308	810.2463
**Egypt**	595.9837*	3667.1669	974.6546	580.3547	355.6656	4462.9528	1178.5203	778.3899
**Greece**	588.9837*	3374.8667	948.9745	585.8479	355.6656	41818.2278	1386.3481	803.9952
Hungary	565.9839	3407.6420	944.8847	594.4351	355.6656	77456.9746	1392.4886	847.0749
**India**	595.9836*	3453.5846	943.7163	585.6860	355.6657	4900.7274	1106.5492	806.6565
Indonesia	565.9838	3598.5045	946.9344	587.4744	355.6657	7597.8481	1215.3103	845.4528
Korea	565.9839	3872.8018	984.5980	603.1732	355.6656	87214.6315	1402.1667	855.8950
**Kuwait**	597.9837*	3832.4529	949.3820	587.5247	355.6656	4924.3725	1162.5570	812.3944
Malaysia	565.9843	4224.3182	983.0466	604.5276	355.6657	1764.2725	1214.4106	773.7504
**Mexico**	900.9843*	3375.2399	926.9907	603.4964	355.6656	11854.8331	1199.4316	772.8167
Peru	565.9830	3366.3168	936.7092	595.2261	355.6656	4820.8405	1123.1589	812.8708
Philippines	565.9833	4181.2511	967.3380	593.8415	355.6656	88655.9364	1409.1255	816.4420
Poland	565.9837	4852.9885	977.6153	587.1007	355.6656	78998.9626	1162.7511	807.4303
Qatar	565.9908	3486.4058	922.0948	602.0380	355.6654	1804.2154	1258.8576	813.3655
Saudi Arabia	565.9838	3485.0640	941.3370	599.9448	355.6656	4898.6249	1113.0103	812.7980
South Africa	565.9852	3848.3770	972.9072	604.9485	355.6656	19706.4951	1170.5992	829.9784
Thailand	565.9838	3891.7516	987.9217	601.9925	355.6656	4893.6159	1160.9646	812.9580
**Turkiye**	599.9841*	3478.3367	942.8258	583.2738	355.6713	4959.8749	1159.0802	837.3508
United Arab Emirates	565.9838	3546.4875	943.0716	593.8109	355.6656	4956.4324	1162.0672	813.4123

Bootstrap critical values are obtained from 10.000 iterations

* significant at 10%

**Fig. 3:**
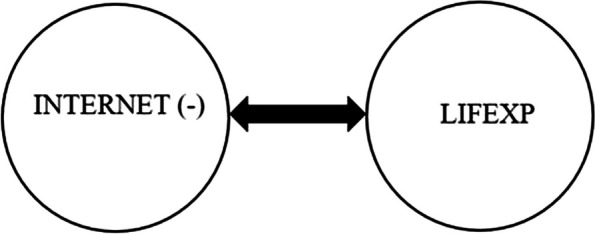
Results of asymmetric bootstrap Granger causality test between INTERNET and LIFEXP (-)

Thirdly, the causality between INTERNET and LIFEXP with negative shocks is tested, and the results in Table 10 indicate a bidirectional causal relationship between INTERNET and LIFEXP in all countries. In other words, a negative shock in INTERNET is a Granger cause of decreases in LIFEXP, and a negative shock in LIFEXP is a Granger cause of decreases in INTERNET (Fig. 3).

**Table 10:** Results of asymmetric bootstrap Granger causality test

Country	INTERNET ↛ LIFEXP (-)				LIFEXP ↛ INTERNET (-)			
	Wald. Stat.	Bootstrap Critical Values			Wald. Stat.	Bootstrap Critical Values		
		1%	5%	10%		1%	5%	10%
**Brazil**	601.8000*	1885.5791	908.2627	511.3923	673.6025*	2164.6313	1335.5862	647.3300
**Chile**	608.8014*	1880.4118	908.3144	559.2154	659.6025*	3413.0624	1336.2877	647.3560
**China**	602.7589*	1846.5634	908.2799	511.2636	782.2867*	2251.6898	1136.7039	624.0494
**Colombia**	701.4623*	1877.3001	893.4974	511.2455	881.0000*	2210.2730	1326.3740	646.9940
**Czechia**	600.2706*	1879.7905	802.0624	511.1512	985.5107*	1859.9582	1336.7869	644.1122
**Egypt**	503.5255*	1883.4063	905.2983	484.1079	980.4717*	1855.7709	1277.7051	632.6240
**Greece**	602.6743*	3046.5859	921.1395	559.6518	657.4191*	2165.7440	1343.0349	647.2314
**Hungary**	709.3765*	1888.5431	799.2246	484.4059	886.8280*	4263.0216	1677.2190	804.5299
**India**	705.7624*	2053.5983	961.2498	559.6407	989.5906*	13460.1502	1676.1072	821.5879
**Indonesia**	601.7881*	1883.0376	907.9066	511.2717	689.5915*	2227.9145	1229.7021	647.0472
**Korea**	501.7827*	1842.9369	780.0236	484.0532	865.9756*	2225.4575	1239.5612	646.9657
**Kuwait**	701.7699*	1543.3128	896.8213	511.2010	687.9575*	2222.3936	1325.2219	633.7524
**Malaysia**	600.9265*	1885.4935	907.9935	511.1983	775.3794*	2221.6877	1126.7975	623.2690
**Mexico**	682.7273*	1877.3300	800.5795	511.2708	990.6249*	19136.2496	1588.6225	819.7078
**Peru**	697.5982*	1849.6077	906.4144	511.1488	720.5560*	2284.1760	1220.0038	624.0953
**Philippines**	704.5323*	1852.4743	706.3812	511.0007	700.4931*	2280.3157	1340.3830	633.5815
**Poland**	508.7952*	1867.4580	883.1288	480.1159	699.4213*	2275.5458	1337.5934	646.4503
**Qatar**	604.6290*	1853.7777	829.5298	511.3587	690.5805*	2286.9815	1338.1316	647.0331
**Saudi Arabia**	601.7393*	1849.6497	891.2299	483.7114	689.5090*	2301.8433	1335.7199	647.0803
**South Africa**	708.5846*	1887.3339	908.5727	511.3519	672.6127*	2195.3396	1346.5409	632.6496
**Thailand**	794.8346*	1544.8064	908.7169	511.3496	788.6187*	2221.0260	1345.8264	646.9839
**Turkiye**	697.6750*	1889.9811	802.8486	511.1471	981.1980*	1965.5648	1330.7510	821.3027
**United Arab Emirates**	709.0836*	1888.7375	852.9333	511.2591	688.4297*	2226.6251	1334.7541	647.2732

* significant at 10%

**Fig. 4:**
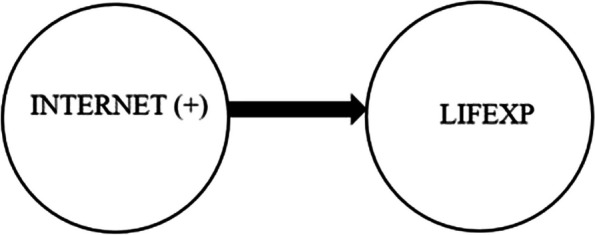
Results of asymmetric bootstrap Granger causality test between INTERNET and LIFEXP (+)

Last, the causality between INTERNET and LIFEXP with positive shocks is tested, and the results in Table 11 indicate a one-way causal relationship from INTERNET to LIFEXP in all countries. In other words, a positive shock in INTERNET is a Granger cause of increases in LIFEXP. However, positive shocks from LIFEXP do not significantly influence the INTERNET (Fig. 4).

**Table 11:** Results of asymmetric bootstrap Granger causality test

Countries	INTERNET ↛ LIFEXP (+)				INTERNET ↛ LIFEXP (+)			
	Wald. Stat.	Bootstrap Critical Values			Wald. Stat.	Bootstrap Critical Values		
		1%	5%	10%		1%	5%	10%
Brazil	1604.3982***	901.6496	712.5125	505.8883	360.2541	5227.3093	1374.3320	673.3605
Chile	1604.3798***	902.6157	712.1273	505.8564	360.2536	6423.5978	1527.7549	685.1035
China	1604.3977***	902.4996	581.5654	500.5946	360.2537	5230.1144	1355.4228	668.9929
Colombia	1604.3893***	901.4093	703.6941	501.2596	360.2541	9682.1689	1532.5747	684.8797
Czechia	1604.3916***	902.0091	710.4013	505.4409	360.2539	9840.3639	1533.3168	661.2260
Egypt	1604.4084***	902.6015	711.8036	505.5609	360.2535	7824.9889	1525.9040	685.3332
Greece	1604.3818***	869.3635	711.6893	505.6365	360.2540	10127.1222	1452.8678	674.8826
Hungary	1604.3933***	901.2638	712.0846	505.8536	360.2540	4991.4586	1365.8051	666.3999
India	1604.3942***	900.1705	711.9872	492.8784	360.2538	4616.2783	1415.1645	666.4301
Indonesia	1604.3875***	893.0953	712.5602	501.3657	360.2496	5283.9756	1427.0975	659.6398
Korea	1604.3596***	860.7035	709.6218	505.9109	360.2535	5303.8168	1420.3056	583.2947
Kuwait	1604.3942***	902.4183	703.7067	505.6608	360.2534	8968.4190	1519.5099	684.7390
Malaysia	1604.3963***	902.5492	712.0071	505.7807	360.2536	4477.4889	1406.8260	646.8198
Mexico	1604.0047***	837.5743	712.5611	505.8260	360.2536	5537.2579	1420.8017	673.9002
Peru	1604.3927***	900.5327	604.8136	501.4270	360.2535	5091.0023	1423.2597	686.4929
Philippines	1604.3999***	899.4409	562.5920	474.9731	360.2525	5514.0054	1366.4489	666.1510
Poland	1604.3902***	897.6414	712.5377	505.8737	360.2502	5090.5121	1322.0162	660.8177
Qatar	1604.3893***	902.3358	594.5828	505.7427	360.2499	5287.8752	1410.6584	596.1401
Saudi Arabia	1604.3956***	1095.9186	794.3599	516.0852	360.2533	4932.5356	1425.2956	670.1560
South Africa	1604.3960***	893.7262	704.3965	505.9311	360.2533	5308.3014	1381.9825	595.4400
Thailand	1604.3042***	1899.7406	779.2146	519.6091	360.2535	9337.6716	1522.5854	684.6008
Turkiye	1604.3938***	902.2169	712.6304	492.7523	360.2536	9956.0164	1523.0617	679.2175
United Arab Emirates	1604.3909***	902.5361	606.0280	505.4038	360.2535	9961.0716	1421.1065	685.9879

* significant at 10%

## Discussion

ICT theoretically can influence life expectancy via various positive and negative channels such as access and sharing of information about health, preventative health care, healthy nutrition, epidemics, economic growth and development, unemployment, education, environment, green technological progress, energy use, insufficient physical activity, digital addiction, and cyber security problems in the light of related theoretical and empirical literature. Therefore, the net impact of ICT penetration on life expectancy can differ depending on which factors outweigh the others. On the other hand, life expectancy can also affect ICT penetration because people have more time to use and develop the ICT.

Our symmetric causality analysis uncovers a feedback interaction between mobile subscriptions and life expectancy. In other words, both mobile subscriptions and life expectancy affect each other. However, the asymmetric causality test results indicate that increases in mobile subscriptions significantly cause increases in life expectancy in China, Colombia, Czechia, Egypt, Greece, India, Kuwait, Mexico, and Turkiye. Therefore, our findings are compatible with the theoretical considerations and results of Lee and Kim [31], Majeed and Khan [32], Mlambo et al. [36], and Wang et al. [41]. In conclusion, mobile subscriptions are expected to influence life expectancy via multiple channels described in the theoretical and empirical literature.

Our symmetric causality analysis uncovers that internet usage does not significantly affect life expectancy, but life expectancy has a significant effect on internet usage. On the other hand, the results of the asymmetric causality test reveal that internet usage significantly influences life expectancy in the case of both positive and negative shocks in internet usage, which is compatible with theoretical considerations. This finding also verified the asymmetric causality test’s importance in uncovering the hidden interaction between two variables. Furthermore, most of the empirical studies, including Mimbi and Bankole [30], Lee and Kim [31], Alzaid et al. [33], Zhang et al. [38], Byaro et al. [39], and Wang et al. [41] have analyzed the interaction between ICT proxied by internet usage and life expectancy and discovered a significant influence of the Internet on life expectancy through disseminating of health-related information, easing the healthcare services, increasing the early detection and treatment of diseases, and improving the effective and efficient use of clinic time.

## Conclusion

Life expectancy is a crucial result indicator of multiple sustainable development goals such as no poverty, zero hunger, good health and well-being, quality education, clean water and sanitation, decent work and economic growth. Therefore, improvements in life expectancy also mean that the relevant societies also progress in overall sustainable development. In this regard, detecting factors underlying sustainable development has become crucial. This study investigates the interaction between ICT indicators of mobile subscription and internet usage and life expectancy through symmetric and asymmetric causality tests.

In the related empirical literature, the researchers have usually represented the ICT by internet usage and mobile subscriptions. However, many social, cultural, demographic, and economic variables have the potential to impact life expectancy. This study centres upon the two-way interplay between ICT indicators and life expectancy by excluding the other possible variables in the analyses. Therefore, our findings are helpful for the nexus between ICT and life expectancy, but the ignored variables can influence the relationship between ICT indicators and life expectancy. Furthermore, the study accepts that all variables are endogenous because they are determined within the model through the causality test. Last, the presence of ICT indicators limits us to conduct the study for the 1997–2020 duration.

The findings of the symmetric causality test uncover that both ICT indicators significantly influence life expectancy when analyzing the causality between two ICT indicators and life expectancy, but mobile subscriptions are the driving factor. On the other hand, the causality test reveals a bidirectional causal relationship between mobile subscriptions and life expectancy and a unidirectional causal interaction between life expectancy and internet usage.

On the other side, the results of the asymmetric causality test uncover a unidirectional causal relationship between mobile subscriptions and life expectancy in China, Colombia, Czechia, Egypt, Greece, India, Kuwait, and Turkiye in case of positive shocks from both variables. Furthermore, a bidirectional causal relationship exists between internet usage and life expectancy in all countries in case of negative shocks from both variables. Lastly, a one-way causal relationship between internet usage and life expectancy in all countries is uncovered in case of positive shocks from internet usage.

Based on the empirical findings of this paper, three significant policy suggestions are made to improve life expectancy through ICT:

First, public and private sectors should encourage ICT infrastructure and ICT services, such as e-health, healthy nutrition, preventative health care, e-government, and e-learning, through financial and regulatory incentives such as tax deductions and cash support. Secondly, education and training programs should be designed to improve digital literacy and ICT adoption. Thirdly, financial incentives and regulations should encourage ICT technologies that support the efficient use of natural resources such as energy and water and sustainable cities.

This research focuses on the nexus between ICT indicators and life expectancy. However, economic, social, cultural, and demographic variables also can impact the nexus between ICT indicators and life expectancy. Therefore, future studies can investigate the impact of the ignored variables, such as educational attainment and cultural differences, on the nexus between ICT and life expectancy.

## Data Availability

The data used in the research is obtained from open-access databases of the UNDP and the World Bank, and further inquiries can be directed to the corresponding author/s.

## Abbreviations

ARDL: Autoregressive Distributed Lag
BRICS-T: Brazil, Russian Federation, India, China, South Africa, Turkiye
CIPS: Cross-sectionally augmented Im-Pesaran-Shin
HPJ: Half-panel jackknife
ICT: Information and communication technologies
JKS: Juodis-Karavias-Sarafidis
LM: Lagrange Multiplier
LM CD: Lagrange Multiplier Cross-sectional Dependence
S.D.G.: Sustainable Development Goals
UNDP: United Nations Development Programme
